# Novel Environmental Niches for *Candida auris*: Isolation from a Coastal Habitat in Colombia

**DOI:** 10.3390/jof8070748

**Published:** 2022-07-19

**Authors:** Patricia Escandón

**Affiliations:** Grupo de Microbiología, Instituto Nacional de Salud, Bogotá 111321, Colombia; pescandon@ins.gov.co; Tel.: +57-1-2207700

**Keywords:** *Candida auris*, climate change, Colombia, environment

## Abstract

Climate change has been proposed as a set of factors that affect the frequency, distribution, and morbimortality of many infectious diseases, in which mycosis has also been impacted. Most fungi have the ability to cause disease in mammalian hosts as a result of their competitive fitness advantages that allow adaptation to diverse ecological niches. *Candida auris* has burst in the infectious disease scenario, and it has been hypothesized that a combination of stress adaptation and biotic predation has driven this fungus in the evolution of thermotolerance and halotolerance mechanisms to adapt to different environmental niches, which have resulted in the capacity to cross the thermal infection barrier in humans. Consequently, the isolation of *C. auris* from estuaries in Colombia adds to the evidence that suggests that this fungus existed in the environment previously to being recognized as a human pathogen, and promotes the need for further investigations to identify additional ecological niches.

Rhijin et al. [1] recently suggested in a review published in JOF, and previously exposed by Casadevall et al. [2], that climate change has imposed an impact on several aspects, including the epidemiological landscape of pathogens, in which fungi play an important role as microorganisms with the capability of rapidly adapting to changing environments, thus resulting in newly emerging pathogens such as *Candida auris*.

Sporadic cases of *C. auris* date from 2009 in Japan [3], but the number of infections and colonizations detonated two years later, with the appearance of this pathogen in different hospital settings, which has emerged independently and simultaneously in most parts of the world with different evolutionary histories and genome-wide patterns of variation [4]. *C. auris* has imposed challenges to the scientific community because of its particular characteristics: causes serious infections, especially in ill patients; is frequently and unnervingly resistant to common antifungals drugs; identification by some of the laboratory tests available is inexact; and because of its ease of propagation between hospital settings and patients, it commonly causes outbreaks, thus becoming a major public health problem.

Until recently, there was no evidence of the origin of *C. auris* as an environmental fungus since the description of having its own environmental niche was unknown. Some authors had proposed several hypotheses for the emerging of this fungus as a human pathogen, including the human-induced global warming [2] and impact of environmental and human populations changes that have pressured *C. auris* to evolve into a more invasive and resistant microorganism [5], which, in turn, have resulted in a pathogen capable of breaking through the thermal infection barrier of mammals, with the consequences which are now evident. The thermotolerance of *C. auris* has been also proposed to be a characteristic that has allowed birds to transport the fungus across the globe to rural areas where humans and birds are in constant contact [2]. The isolation of *C. auris* from a marine habitat, specifically from a salt marsh and sandy beach in the Coastal Wetlands of the Andaman Islands in India [6], emphasizes the hypothesis that, prior to its recognition as a human pathogen, *C. auris* existed as an environmental fungus. 

In Colombia, *C. auris* has been retrospectively identified in the clinical scenarios since 2015, when an unusual number of *C. haemulonii* bloodstream infection cases were reported, which were later confirmed as being *C. auris*. As of this finding, in 2016, the National Institute of Health in Colombia (INS) issued a national alert on the emergence of *C. auris*, leading to the implementation of the national surveillance of this emerging multi-drug-resistant fungus. This ongoing surveillance has resulted in 1720 *C. auris* cases, represented by 23% of colonization cases and 77% of infections in patients living in 62% of the national territory [7], with low multi-drug resistance (12%) and no pan-resistant isolates identified. Although in Colombia several outbreak investigations have been carried out [8,9], and *C. auris* has been isolated from the hospital settings environment, the lack of information on the possible environmental niche harboring the fungus in the national territory persisted. As part of routine activities accomplished in other research scenarios, 49 water samples were collected in the year 2018 in several marine and estuary bodies of waters, collecting approximately 100 mL of water from a depth of 30 cm. Samples were transported to the Microbiology Laboratory of INS for further processing in a salt Sabouraud Dextrose selective broth [10]. Briefly, samples were vortexed in 30 mL of phosphate buffer saline (PBS 1X) with Tween 1X, and the supernatant transferred to a 50 mL conical tube and centrifuged at 20,000× *g* for 15 min. The resulting precipitate, in a volume of 1 mL, was transferred to a tube containing Sabouraud Dextrose broth supplemented with 10% NaCl, incubated at 40 °C for 48 h with constant agitation of 5 g. A volume of 100 μL was streaked onto salt Sabouraud Dextrose plates and incubated at 30 °C for 7 days. Yeast isolates recovered from the selective plates were typed using the MALDI-TOF MS technology (BD™ Bruker MALDI Biotyper, Bremen, Germany). This sampling and processing of samples in search of the habitat of *C. auris* resulted in the isolation of this pathogen from estuaries, a mixture of fresh water draining from the land and salty seawater located in the northwest of Colombia, about 254 miles from Bogotá, the capital city, at a latitude of 6.105444 and longitude of −77.423333, with a mean temperature of 28 °C and a relative humidity of 70%. One (2%) positive sample was identified, from which 2 CFU/mL of *C. auris* were recovered, was characterized by having a superficial temperature of 25.2 °C, low salinity and pH values than ranged between 7.4 and 8.2. The habitat is characterized by the presence of diverse fauna and flora, with not extensive human activity in the surroundings. *C. auris* was identified, after growth in high salinity Sabouraud dextrose agar incubated at 40 °C, by matrix-assisted laser desorption ionization–time of flight mass spectrometry (MALDI-TOF MS) (Bruker Biotyper OC v.3.1; Daltonics, Bremen, Germany) and PCR species specific [11]. Antifungal susceptibility testing by CLSI broth microdilution method [12] to amphotericin B, fluconazol, voriconazol and anidulafungin showed low MICs to the antifungal drugs tested (0.5, 2.0, 0.25 and 0.25 μg/mL, respectively).

Because fungal thermal tolerance at mammalian temperatures is essential for virulence and infections in humans, it is interesting to consider the suggestions made by Robert et al. [13] mentioning that fungi phylogenetically closely related to pathogenic fungi may acquire pathogenic potential as a result of an increase in thermal tolerance result of the adaptation to global warming and/or evolution. Reports have been made on the isolation from seawater and other coastal habitats for *C. haemulonii, C. pseudointermedia, C. intermedia* and *C. torresii*, all belonging to the Metschnikowiaceae family, which may support the hypothesis previously mentioned [13,14,15].

As stated by Rhajin et al., climate change impacts all classes of scenarios, in which host-pathogens interactions are not the exception [1]. Particularly, for *C. auris*, stress adaptation mechanisms and biological interactions with other organisms have forced this fungus to evolve in thermotolerance and halotolerance mechanisms [16]. As the environment is continuously undergoing changes, natural or imposed by humans, we will be more liable to be exposed to fungi and other potential pathogens we never imagined to exist. Whether *C. auris* emergence is possibly the result of climate change resulting in a complex interaction between host–pathogen and environmental factors, more research needs to be done to understand the behavior of this efficient pathogen.

This first isolation of *C. auris* from an environmental source in Colombia has an impact on the epidemiology of the disease in our country, suggesting bodies of water as a natural habitat for this pathogen, as potential infection sources for acquiring human infections. Whole-Genome Sequencing of the recovered isolate in Colombia needs to be performed as a tool that will assign a phylogenetic clade to this environmental strain of *C. auris*, and intensive samplings need to be done in order to identify other natural habitats for *C. auris*, as well as to contribute to the genomic surveillance of this pathogen.

## References

[1] van Rhijn N., Bromley M. (2021). The consequences of our changing environment on life threatening and debilitating fungal diseases in humans. J. Fungi.

[2] Casadevall A., Kontoyiannis D.P., Robert V. (2019). On the Emergence of *Candida auris*: Climate Change, Azoles, Swamps, and Birds. mBio.

[3] Satoh K., Makimura K., Hasumi Y., Nishiyama Y., Uchida K., Yamaguchi H. (2009). *Candida auris* sp. nov., a novel asco-mycetous yeast isolated from the external ear canal of an inpatient in a Japanese hospital. Microbiol. Immunol..

[4] Lockhart S.R., Etienne K.A., Vallabhaneni S., Farooqi J., Chowdhary A., Govender N.P., Colombo A.L., Calvo B., Cuomo C.A., Desjardins C.A. (2017). Simultaneous Emergence of Multidrug-Resistant *Candida auris* on 3 Continents Confirmed by Whole-Genome Sequencing and Epidemiological Analyses. Clin. Infect. Dis..

[5] Sekizuka T., Iguchi S., Umeyama T., Inamine Y., Makimura K. (2019). Clade II *Candida auris* possess genomic structural variations related to an ancestral strain. PLoS ONE.

[6] Arora P., Singh P., Wang Y., Yadav A., Pawar K., Singh A., Padmavati G., Xu J., Chowdhary A. (2021). Environmental Isolation of *Candida auris* from the Coastal Wetlands of Andaman Islands, India. mBio.

[7] Escandón P., Cáceres D.H., Lizarazo D., Lockhart S.R., Lyman M., Duarte C. (2021). Laboratory-based surveillance of *Candida auris* in Colombia, 2016–2020. Mycoses.

[8] Caceres D.H., Rivera S.M., Armstrong P.A., Escandon P., Chow N.A., Ovalle M.V., Díaz J., Derado G., Salcedo S., Berrio I. (2020). Case–Case Comparison of *Candida auris* Versus Other Candida Species Bloodstream Infections: Results of an Outbreak Investigation in Colombia. Mycopathol. Mycol. Appl..

[9] Armstrong P.A., Rivera S.M., Escandon P., Caceres D.H., Chow N., Stuckey M.J., Díaz J., Gomez A., Vélez N., Espinosa-Bode A. (2019). Hospital-Associated Multicenter Outbreak of Emerging Fungus *Candida auris*, Colombia, 2016. Emerg. Infect. Dis..

[10] Welsh R.M., Bentz M.L., Shams A., Houston H., Lyons A., Rose L.J., Litvintseva A. (2017). Survival, Persistence, and Isolation of the Emerging Multidrug-Resistant Pathogenic Yeast *Candida auris* on a Plastic Healthcare Surface. J. Clin. Microbiol..

[11] Kordalewska M., Zhao Y., Lockhart S.R., Chowdhary A., Berrio I., Perlin D.S. (2017). Rapid and Accurate Molecular Identification of the Emerging Multidrug-Resistant Pathogen *Candida auris*. J. Clin. Microbiol..

[12] CLSI M60 Performance Standards for Antifungal Susceptibility Testing of Yeasts, 1st 157 Edition 2017. http://clsi.org.

[13] Robert V., Cardinali G., Casadevall A. (2015). Distribution and impact of yeast thermal tolerance permissive for mammalian infection. BMC Biol..

[14] Kolipinski M.C., Van Uden N. (1962). *Torulopsis haemulonii* nov. spec. a yeast from the Atlantic ocean. Antonie Van Leeuwenhoek.

[15] Buzzini P., Lachance M.A., Yurkov A. (2017). Yeasts in Natural Ecosystems: Diversity.

[16] Casadevall A., Fu M.S., Guimaraes A.J., Albuquerque P. (2019). The ‘Amoeboid Predator-Fungal Animal Virulence’ Hypothesis. J. Fungi.

